# Pharmacology of Antiviral Drugs

**DOI:** 10.3390/v17040459

**Published:** 2025-03-24

**Authors:** David E. Martin

**Affiliations:** TrippBio, Inc., Jacksonville, FL 32256, USA; davidmartin@trippbio.com

Antiviral drug therapy has significantly evolved over the past four decades, partly driven by improved understanding of viral replication mechanisms but also advances in pharmacology and formulation technologies. Historically, viral infections posed substantial therapeutic challenges due to the limited availability of effective antiviral agents and the rapid emergence of drug resistance. Unlike bacterial infections, which can often be effectively managed with antibiotics, viruses replicate intracellularly using host cellular machinery, complicating the development of selective antiviral therapies. Early antiviral drug discovery efforts were further hindered by inadequate knowledge about viral life cycles and limited technological capabilities for drug screening and formulation development. However, significant breakthroughs in molecular biology, virology, medicinal chemistry, pharmacology, and pharmacokinetics have progressively transformed the landscape of antiviral therapeutics.

One of the best examples of this can be seen in the treatment of human immunodeficiency virus (HIV) infection. HIV emerged in the early 1980s as a global epidemic characterized by rapid disease progression and high mortality rates. Initially, no effective treatments existed for the treatment of HIV infection, which prompted unprecedented research efforts and collaborations between academia, industry, regulatory authorities, and patient advocacy groups. This collective effort led to rapid advancements in antiviral drug discovery and development, ultimately revolutionizing HIV treatment paradigms from high-pill-burden regimens to simplified single-tablet formulations and now long-acting injectable therapies that dramatically improve patient adherence and quality of life.

These advancements began with the approval of azidothymidine (AZT, zidovudine), marking a pivotal turning point in antiviral pharmacology. AZT was the first antiretroviral drug approved by the U.S FDA in 1987 for the treatment of HIV infection [1]. Initially developed as an anti-cancer treatment in 1964, AZT was later discovered to inhibit HIV replication by selectively targeting HIV’s reverse transcriptase enzyme [1]. This enzyme is essential for converting viral RNA into DNA, a critical step in HIV’s life cycle, and was the first viral target successfully exploited as an effective therapy for HIV infection. At its initial approval, AZT required frequent dosing every four hours and came with significant side effects, including anemia and myositis due to mitochondrial toxicity.

## 1. Progression to Combination Therapies and HAART

Early single-drug therapies like AZT quickly led to drug resistance, prompting the development of combination therapies. By 1996, triple-drug regimens emerged, commonly referred to as highly active antiretroviral therapy (HAART), combining drugs from different classes to durably suppress HIV replication and minimize resistance. Despite their efficacy, these early HAART regimens were burdensome for patients, requiring strict adherence to complex dosing schedules involving dozens of tablets, dietary restrictions, and high fluid intake.

## 2. Fixed-Dose Combinations and Single-Tablet Regimens

Recognizing adherence challenges with high-pill-burden regimens, pharmaceutical companies collaborated to develop fixed-dose combination therapies. In 2006, Atripla^®^ became the first complete single-tablet regimen (STR), combining Bristol-Myers Squibb’s efavirenz with Gilead’s emtricitabine and tenofovir disoproxil fumarate [2]. This marked a significant advance towards simplified HIV treatment (i.e., one pill once a day) by improving adherence and patient quality of life.

## 3. Long-Acting Antiviral Formulations

More recently, the development of long-acting injectable formulations designed to dramatically reduce dosing frequency through enhanced pharmacokinetic properties has gained regulatory approvals and widespread acceptance [3]. These formulations utilize technologies such as crystalline nanoparticle suspensions or subcutaneous depot injections that slowly release active drugs into circulation over extended periods. This sustained release maintains therapeutic drug concentrations over weeks or months, significantly improving adherence and convenience. The first approval was for Cabenuva (cabotegravir + rilpivirine), initially approved in 2021 as a monthly intramuscular injection but later extended to bi-monthly dosing [4]. In 2022, lenacapavir was approved as a twice-yearly injection, with more recent data suggesting that a once-yearly injection may completely prevent HIV transmission when used prophylactically [5].

## 4. Future Directions

The success of long-acting antivirals in HIV treatment and prophylaxis should be viewed as the proof of concept that this approach can gain regulatory approval and acceptance within the target population. There are a variety of viral infections that could benefit from this approach for both treatment and prophylaxis.

For example, treatment of chronic hepatitis B infection requires lifelong daily dosing due to the inability of current drugs to fully eliminate the virus; thus, ultra-long-acting antivirals could significantly improve adherence and minimize risks associated with HBV reactivation [6]. Similarly, long-acting antivirals for HCV could revolutionize treatment by enabling “test-treat-cure” strategies with single-shot administration shortly after diagnosis [7].

Other respiratory viruses such as influenza and respiratory syncytial virus (RSV) could benefit greatly from long-acting antiviral prophylaxis due to their seasonal nature and adherence challenges associated with frequent oral dosing. Similarly, Ebola virus disease outbreaks could be effectively managed with prophylactic long-acting antivirals that provide sustained protection during epidemic periods.

In an ideal world, many of these viral infections would be prevented with vaccinations, but given the “vaccine hesitancy” that has taken hold over the last few years, an effective drug-based prophylactic approach might be more acceptable and could be a viable alternative.

## 5. Incorporating Long-Acting Approaches into Drug Discovery Programs

Given these promising developments across multiple viral diseases, pharmaceutical companies should strategically incorporate long-acting formulation approaches into their antiviral drug discovery programs by enacting the following:

- Investing in advanced formulation technologies such as crystalline nanoparticle suspensions and depot injection platforms;
- Prioritizing compounds with favorable pharmacokinetic profiles suitable for prolonged release;
- Exploring broad-spectrum antiviral candidates that target conserved viral replication mechanisms across multiple viruses, including both direct-acting and host-directed antivirals;
- Collaborating across industry sectors and academic institutions to accelerate translation from preclinical research into clinical development;
- Addressing potential challenges such as injection-site reactions and ensuring safety profiles are optimized for prolonged exposure;
- Conducting targeted clinical trials focused on patient populations with adherence challenges and regions lacking healthcare infrastructure where regular medication adherence is difficult [1];
- Developing regulatory strategies that facilitate rapid approval pathways for unmet medical needs such as HDV infection and emerging infectious diseases.

By proactively incorporating these strategies into antiviral drug discovery programs, pharmaceutical companies can accelerate development timelines and effectively address global health challenges posed by chronic viral infections and emerging epidemics.
